# Adherence to dietary practices recommended by the Dietary Guidelines for the Brazilian Population among people with obesity: baseline of a community trial carried out at the Health Fitness Program in Belo Horizonte, Brazil, 2022-2023

**DOI:** 10.1590/S2237-96222024v34e20240287.en

**Published:** 2025-04-28

**Authors:** Mariana Souza Lopes, Vitória Marli Serafim Moreira Silva, Nathália Luíza Ferreira, Maria Cecília Ramos de Carvalho, Patrícia Pinheiro de Freitas, Aline Cristine Souza Lopes

**Affiliations:** 1 Universidade Federal da Paraíba, João Pessoa, Paraíba, Brazil; 2 Universidade Federal de Minas Gerais, Belo Horizonte, Minas Gerais, Brazil; 3 Universidade Federal de Lavras, Lavras, Minas Gerais, Brazil; 4 Universidade Federal dos Vales do Jequitinhonha e Mucuri, Diamantina, Minas Gerais, Brazil

**Keywords:** Observational Studies, Food Guides, Obesity, Sociodemographic Factors, Primary Health Care, Estudios Observacionales, Guías Alimentarias, Obesidad, Factores Sociodemográficos, Atención Primaria de Salud

## Abstract

**Objective:**

To analyze sociodemographic and health factors associated with high levels of adherence to dietary practices recommended by the *Dietary Guidelines for the Brazilian Population* among people with obesity taking part in the Health Fitness Program in Belo Horizonte, Minas Gerais, Brazil.

**Methods:**

This is a cross-sectional study with a representative sample of Health Fitness Program participants with obesity who were ≥20 years old. Sociodemographic and health data were collected, and the adherence to the Dietary Guidelines scale was assessed, classified as: low (<32 points), medium (32-41 points) and high (>41 points). Multivariate logistic regression was performed.

**Results:**

In all, 1,109 individuals participated, the majority of whom were women (92.5%) and had at least one chronic disease (85.8%). The majority self-rated their health status as very good/good (62.2%), 62.8% had hypertension and 68.0% had Class 1 Obesity. 46.0% had good levels of adherence to the Dietary Guidelines. After adjustment, high levels of adherence were associated with being elderly (odds ratio [OR] 1.47; 95%CI 1.1; 2.0), having higher income (OR 0.58; 95%CI 0.41; 0.81) and better self-rated health (regular: OR 0.73; 95%CI 0.55; 0.96; poor/very poor: OR 0.26; 95%CI 0.13; 0.52).

**Conclusion:**

High levels of adherence to the Dietary Guidelines were associated with stage of life, better income and positive self-rated health, highlighting the need to target efforts towards adults and vulnerable groups

## Resumen

**Objetivo:**

Analisar os fatores sociodemográficos e de saúde associados à alta adesão às práticas alimentares recomendadas pelo *Guia Alimentar para População Brasileira* entre usuários com obesidade do Programa Academia da Saúde de Belo Horizonte, Minas Gerais.

**Métodos:**

Estudo transversal em amostra representativa do Programa Academia da Saúde com participantes com obesidade e idade ≥20 anos. Coletou-se dados sociodemográficos e de saúde, e aplicou a escala de adesão ao Guia Alimentar, classificada em: baixa (<32 pontos), média (32-41 pontos) e alta (>41 pontos). Realizou-se regressão logística multivariada.

**Resultados:**

Participaram 1.109 indivíduos, sendo a maioria mulheres (92,5%) e com alguma doença crônica (85,8%). Quanto às condições de saúde, a maioria avaliou sua saúde como muito boa/boa (62,2%), 62,8% possuíam Hipertensão e 68,0% obesidade I. Alta adesão ao Guia Alimentar foi de 46,0%. Após ajuste, a alta adesão foi associada a ser idoso [razão de chances (*odds ratio*, OR 1,47; IC95% 1,1; 2,0), ter maior renda (OR 0,58; IC95% 0,41; 0,81) e melhor percepção de saúde (regular: OR 0,73; IC95% 0,55; 0,96; ruim/muito ruim: OR 0,26; IC95% 0,13; 0,52). Conclusão: A alta adesão ao Guia foi associada à fase da vida, melhor renda e autoavaliação positiva da saúde, evidenciando a necessidade de direcionar esforços para adultos e grupos vulneráveis.

## Introduction

The principles and recommendations for adequate and healthy eating for Brazilians are presented in the *Dietary Guidelines for the Brazilian Population*, an internationally recognized document. The Dietary Guidelines assumptions are the right to health and adequate food, and the multiple determinants of eating practices (1). Its principles consider food items and their combinations, as well as the cultural and social dimensions of eating practices. Furthermore, they take into consideration interdependence between food and food systems, valuing different forms of knowledge when formulating dietary recommendations (1).

As the basis for its recommendations on an adequate and healthy diet, the Dietary Guidelines recommend the golden rule “Always prefer unprocessed or minimally processed foods and culinary preparations to ultra-processed foods”, in addition to its covering eating habits and commensality (1). This perspective is innovative and moves forward with the implementation of health, food and nutrition policies (2,3).

Several different materials have been developed in order to support the implementation of the Dietary Guidelines within the Brazilian National Health System (*Sistema Único de Saúde* - SUS). Standing out among them is the “How do you rate what you eat” test, which assesses, in a multidimensional and qualitative way, adherence to the Dietary Guidelines recommendations (4). Studies conducted with adults and elderly individuals identified significant association between sociodemographic characteristics and adherence to the Dietary Guidelines (5,6). However, they did not explore this adherence among individuals with obesity and who were users of Primary Health Care services.

Primary Health Care is a privileged setting for implementing the Dietary Guidelines as it encompasses health promotion, health maintenance and health recovery actions, in addition to its proximity to the community and ability to reach the population (7). However, with regard to specific groups, such as people with obesity, it is recommended that the Dietary Guidelines suggested approaches be adapted. 

According to the Clinical Protocol and Therapeutic Guidelines for Adult Overweight and Obesity (*Protocolo Clínico e Diretrizes Terapêuticas de Sobrepeso e Obesidade em Adultos*), care of people with obesity must include promotion of appropriate and healthy food choices (8). However, to achieve this, it is necessary to investigate eating practices and propose actions that go beyond consumption of food groups and nutrients, encompassing ways of eating and commensality, among other eating practices (1). As such, this article aimed to analyze sociodemographic and health factors associated with high adherence to dietary practices recommended by the Dietary Guidelines among people with obesity taking part in the Health Fitness Program (*Programa Academia da Saúde*) in Belo Horizonte, state of Minas Gerais, Brazil.

## Methods

### Study design

This is a cross-sectional, Screening and Baseline study of two Randomized Controlled Community Trials (*Ensaios Comunitários Controlados Randomizados* - ECCR) conducted with people with obesity taking part in the Health Fitness Program in Belo Horizonte, Minas Gerais, between May 2022 and June 2023, with the Screening and Baseline stages being carried out from May to October 2022. The ECCR were carried out simultaneously to evaluate the effectiveness of collective nutritional interventions for the management of obesity among Health Fitness Program participants, according to the severity of their obesity (9).

### Setting

Belo Horizonte is the capital of the state of Minas Gerais and has approximately 2.3 million inhabitants, making it the sixth most populous city in Brazil and a national reference in Primary Health Care organization (10).

The Health Fitness Program is a Brazilian Primary Health Care health promotion service, in existence since 2011. It offers physical exercise/activity; actions to promote adequate and healthy eating, community mobilization, healthy lifestyles, among others (11). In Belo Horizonte, the service offers regular physical exercise, guided by Physical Education professionals, for people aged ≥18 years old joining the service on spontaneous demand or referred by municipal health services. Currently, the Program operates in more than 80 units in the municipality, distributed across nine administrative regions, serving around 15,000 users (12).

### Study size

A unified sampling process was carried out for the ECCRs through simple random sampling stratified by the nine regions of the municipality. The sampling plan was prepared by an external statistician, without involvement of the researchers. Health Fitness Program units that operate in the morning (main operating shift), that had not carried out nutritional interventions to manage obesity in the last two years and that had a Physical Education professional during the research period were considered eligible (9).

The sample calculation took into consideration 20% prevalence of Class I obesity (Body Mass Index – BMI 30-35kg/m²), a 5% difference in the participants’ body weight between the intervention allocation groups, and an additional 35.0% for possible losses, estimated from previous studies. The final sample consisted of 5,652 individuals distributed across 59 units of the Health Fitness Program, being representative of the health service in the city, with 95.0% confidence and <2.8% margin of error (9).

### Participants

Invitations to take part in Screening were made, at the selected Health Fitness Program units (n=59), to all their service users aged ≥20 years old who took part in Health Fitness Program activities (participation in at least one class in the previous month), excluding pregnant women and individuals with cognitive difficulties that would prevent them from answering the questionnaire (9).

After taking part in the Screening, individuals who were obese (BMI≥30kg/m²); who wished and had availability to participate in groups lasting six months or more; as well as readiness to make changes in order to reduce their weight, according to the stages of change “preparation with high self-efficacy”, “action” or “maintain changes” proposed by the Transtheoretical Model (7), were eligible to participate in the next stage of the study, namely the Baseline (n=1,427). In the Baseline, all those who answered the “How do you rate what you eat?” test (9), which was the response variable of this study, were included. Eighteen participants did not answer the test completely and were excluded. Therefore, after applying the study’s inclusion and exclusion criteria, the final sample was composed of 1,109 individuals with obesity, interest/availability to participate in the following stages of the research, prepared to reduce their body weight and who answered the “How do you rate what you eat?” test.

### Data sources and measurement

Data collection was carried out in two stages: Screening and Baseline. During Screening, an anthropometric assessment was carried out and sociodemographic variables, desire and time to participate in groups, readiness to make changes in order to reduce weight, reported morbidity, self-rated health and participation in the Health Fitness Program were investigated. In the Baseline, carried out immediately after the Screening, the “How do you rate what you eat?” test was administered, having been validated for the Brazilian population with reliability (intraclass correlation coefficient 0.82) (4).

Body weight (kg) was measured in a single take using digital weighing scales with a maximum capacity of 200kg; and height (cm) assessed with the aid of a portable stadiometer, with a scale from 0.35m to 2.13m. Both measurements were carried out in the Screening stage (9).

The instruments used were based on national and international surveys, the research group’s previous experience and validated questionnaires. The interviews were carried out face-to-face, using tablets. Data were collected using the free Epicollect5 application and underwnet consistency analysis. In the event of application instability, data collection was carried out using a printed questionnaire, with subsequent input and consistency analysis (9).

Data collection was carried out during the Health Fitness Program’s opening hours and interviews were scheduled according to the service users’ availability. The team of interviewers included health professionals and undergraduates, supervised by nutritionists and a general supervisor, with support from the research coordinators (9).

### Variables

#### Outcome variable: adherence to dietary practices recommended by the Dietary Guidelines

A 24-item multidimensional scale was used to measure adherence to the Dietary Guidelines. The scale had been previously evaluated by convergent validity and invariance analysis for sex, age and schooling and adequate results subgroups (13). The scale comprises four dimensions: planning, household organization, eating habits and food choice. Each item is followed by a Likert scale with the following response options: strongly disagree; disagree; agree; strongly agree. Values ​​from 0 to 3 are assigned to each item, with the score being computed by the sum of the responses, which can vary from 0 to 72. The first 13 items of the scale are aligned with the Dietary Guidelines recommendations and scored so that the answer “strongly agree” is given 3 points. The final 11 items are contrary to Gudie’s recommendations, being scored inversely, whereby the answer “strongly disagree” is given 3 points (13).

The following cutoff points were used to classify adherence to the Dietary Guidelines recommendations: low (up to 31 points), medium (32-41 points) and high adherence (>41 points) (15). The “low adherence” and “medium adherence” categories were grouped together in the multivariate analysis.

#### Explanatory variables of main interest

The sociodemographic variables investigated were: sex (male; female), stage of life (adult: 20-59 years; elderly: ≥60 years), schooling (years), monthly per capita income tertile and marital status (has a partner; does not have a partner). Per capita income was obtained by taking total family income divided by the number of people living in the household. 

In relation to health, the following were investigated: self-rated health (very good/good; regular; poor/very poor), length of time in the Health Fitness Program (years) and reported morbidities (cardiovascular disease, dyslipidemia, sleep apnea, joint diseases, Type 2 diabetes, hypertension, liver disease and mental illness). The prevalence rates of hypertension and Type 2 diabetes (yes; no) were presented both separately and also together with other comorbidities according to the number of chronic diseases (0; 1; 2; 3 or more). Length of time in the Program was obtained by subtracting the Screening interview date from the Program entry date, obtained from health service records. 

BMI (weight/height²) was used to diagnose obesity, categorized in accordance with World Health Organization guidelines. Severe obesity (yes; no) was identified by adapting the criteria of Ordinance No. 424/2013 for bariatric surgery, whereby severe obesity is considered to be: BMI ≥40 kg/m²; and BMI >35 kg/m² with Type 2 diabetes and/or hypertension, sleep apnea or joint diseases (7).

Readiness for obesity treatment was analyzed according to Obesity Management Group Stratification, as proposed in the “Guidelines on a Collective Approach to Obesity Management in the SUS” (*Instrutivo de Abordagem Coletiva para o Manejo da Obesidade no SUS*) (7). This form of stratification proposes that readiness to make changes in order to treat obesity should be assessed by the availability and desire to participate in groups, and by the stages of behavior change and self-efficacy for weight reduction, according to the Transtheoretical Model. The stages of change were evaluated based on an algorithm proposed by the Ministry of Health, containing reciprocally exclusive responses aimed at identifying the individual’s intention to start making changes in order to reduce their weight and the time elapsed since the beginning of the changes. Self-efficacy consists of the confidence that the individual has to carry out and maintain the behavior change, being measured by a validated scale containing three questions with the following answer options: “not at all confident”; “not very confident”; “moderately confident”; “very confident”; “completely confident”. High self-efficacy was considered to be the presence of at least two questions assessed as very confident or completely confident (15,7).

Individuals who were ready to make changes in order to reduce weight were considered to be those in the “preparation with high self-efficacy”, “action” or “maintain changes” stages (7). 

### Bias

In order to guarantee data control and quality, and adequate internal validity of the study, the entire team was trained in advance for data collection, under the supervision of a health professional, in addition to having manuals and field logistics to guide the data collection activities (9).

### Statistical methods

In order to verify association between the participants’ characteristics and degree of adherence to the dietary practices recommended by the Dietary Guidelines, we used Pearson’s Chi-square statistical test for categorical variables and the Kruskal-Wallis statistical test for numerical variables. 

In order to assess the relationship between high adherence to the Dietary Guidelines (yes; no) and the participants’ sociodemographic and health characteristics, we used multivariate logistic regression, with estimates of odds ratios (OR) and respective 95% confidence intervals (95%CI). The model was adjusted for sex.

The analyses were performed using STATA/SE, with a 5% significance level.

## Results

Of the 1,109 individuals eligible for the study, the majority were women (92.5%), elderly (56.1%) and with more than three years of participation in the Health Fitness Program (66.5%). Regarding health conditions, the majority rated their health as very good/good (62.2%), 62.8% had hypertension and 68.0% had Class I obesity (Table 1).

**Table 1:** Sociodemographic, health and nutritional status characteristics according to degree of adherence to dietary practices recommended by the Dietary Guidelines among people with obesity taking part in the Health Fitness Program. Belo Horizonte, 2022-2023 (n=1,109)

Variables	Total Sample n=1109 (100%)	Adherence to the *Dietary Guidelines for the Brazilian Population*			p-value
		High n=510	Medium n=418	Low n=181	
Sex					0.404^a^
Male	7.5	7.3	8.6	5.5	
Female	92.5	92.7	91.4	94.5	
**Stage of life**					<0.001^a^
Adult	43.9	35.5	45.5	64.1	
Elderly	56.1	64.5	54.5	35.9	
**Marital status**					0.671^a^
Has a partner	56.0	56.9	54.3	57.5	
Does not have a partner	44.0	43.1	45.7	42.5	
**Schooling^c^; median** (P_25_-P_75_)^f^	8.0 (4.0-11.0)	9.0 (4.0-11.0)	8.0 (4.0-11.0)	8.0 (4.0-11.0)	0.298^b^
**Monthly income per capita in BRL** (tertiles)					<0.001^a^
1st tertile (BRL 57-BRL 606)	36.0	29.4	39.5	46.4	
2nd tertile (BRL 625-BRL 1,212)	38.0	39.4	36.1	38.7	
3rd tertile (BRL 1,250-BRL 10,000)	26.0	31.2	24.4	14.9	
**Professional occupation**					<0.001^a^
Housewife	30.2	28.8	30.4	33.7	
Retired	36.2	41.8	34.9	23.2	
Unemployed	4.3	2.6	5.0	7.7	
Other	29.3	26.9	29.7	35.4	
**Self-rated health ^d^**					<0.001^a^
Good/very good	62.2	68.0	58.6	54.1	
Regular	32.4	29.6	34.6	35.4	
Poor/very poor	5.4	2.4	6.7	10.5	
Hypertension	62.8	64.8	63.9	54.7	0.045^a^
Type 2 diabetes	29.9	32.7	29.7	22.2	0.030^a^
**Number of chronic diseases; median** (P_25_-P_75_) ^f^	2.0 (1.0-3.0)	2.0 (1.0-3.0)	2.0 (1.0-3.0)	2.0 (1.0-3.0)	0.325^b^
None	14.2	12.3	15.3	17.1	0.366^a^
1	25.2	26.5	23.7	24.9	
2	25.7	25.9	24.2	28.7	
>3	34.9	35.3	36.8	29.3	
**Obesity class**					0.083^a^
I	68.0	72.1	64.0	66.0	
II	24.8	22.2	27.0	26.8	
III	7.2	5.7	9.0	7.2	
**Severe obesity**					0.230^a^
Not severe	86.0	87.5	85.9	82.3	
Severe	14.0	12.5	14.1	17.7	
**Readiness to treat obesity**					0.496^a^
Yes	62.2	65.5	62.2	58.6	
No	37.8	36.5	37.8	41.4	
**Length of time in the Health Fitness Program** (years)^e^					0.003^a^
1st tertile (0-2.8)	33.5	29.2	34.4	43.4	
2nd tertile (2.9-4.6)	33.2	33.3	32.9	33.5	
3rd tertile (4.7-16.5)	33.3	37.5	32.7	23.1	

^a^Pearson’s Chi-square test; ^b^Kruskal-Wallis test; ^c^4 data missing; ^d^2 data missing; ^e^49 data missing ^f^P25=Percentile 25; P75=Percentile 75.

The final score on the Dietary Guidelines adherence scale ranged from 14 to 66 points, with a median of 41 points (data not shown). The prevalence rate for high adherence was 46.0%, while for medium adherence it was 37.7% and for low adherence it was 16.3% (data not shown).

**Figure 1:**
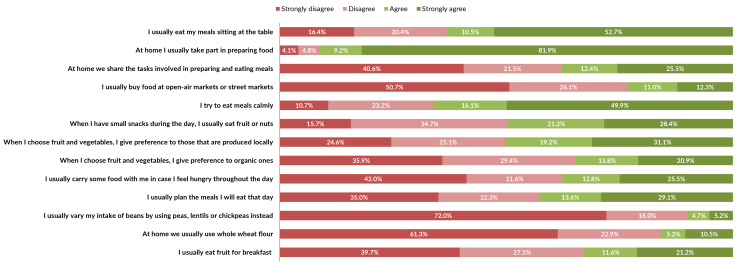
Prevalence of items on the scale of adherence to eating practices aligned with the recommendations of the Dietary Guidelines among people with obesity taking part in the Health Fitness Program. Belo Horizonte, 2022-2023 (n=1,109)

**Figure 2:**
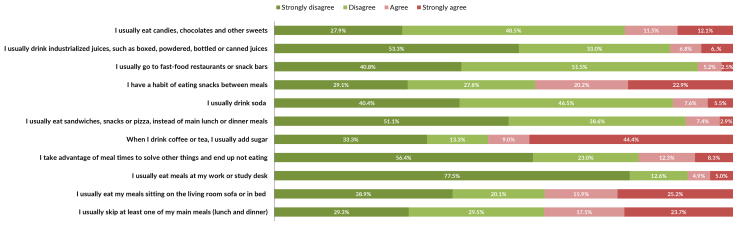
Prevalence of items on the scale of adherence to eating practices not aligned with the recommendations of the Dietary Guidelines among people with obesity taking part in the Health Fitness Program. Belo Horizonte, 2022-2023 (n=1,109)

Figure 1 shows dietary practices that are aligned with the Dietary Guidelines, while Figure 2 shows those that are not. The highest prevalence of positive dietary practices was found for taking part in preparing food at home 91,1% (81.9% - strongly agree + 9.2% - agree) (Figure 1), while the highest prevalence of negative practices was found for drinking coffee or tea sweetened with sugar (53.4%) (Figure 2).

The sociodemographic and health characteristics associated with high adherence to the Dietary Guidelines recommendations were: stage of life, income, self-rated health, self-reported hypertension and Type II diabetes and length of time participating in the Health Fitness Saúde Program (p-value<0.050) (Table 1).

**Figure 3:**
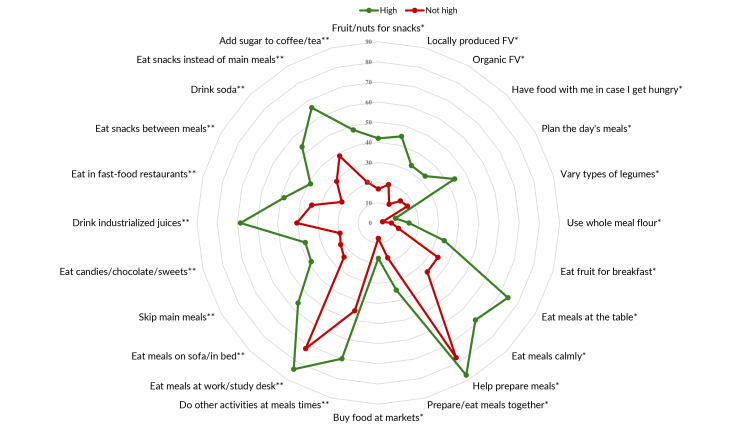
Prevalence of items on the scale of adherence to dietary practices recommended* and not recommended** by the Dietary Guidelines, according to their dimensions, among people with obesity taking part in the Health Fitness Program, Belo Horizonte, 2022-2023 (n=1,109)

Distribution of the scale items according to high adherence to the Dietary Guidelines (yes; no) (Figure 3) showed low prevalence in both groups for “use of wholemeal flour” (15.3% and 6.5%, respectively), “buying food at open-air markets or street markets” (17.6% and 7.7%, in that order) and “varying bean intake by using other legumes” (8.8% and 2.2%, respectively); and greater difference between the groups in relation to eating “meals at work/study desk” (74.3% and 34.2%, in that order). Regarding the dimensions of the scale, greater differences were identified for meal planning (3A), and smaller ones for eating habits (3C). 

**3A:**
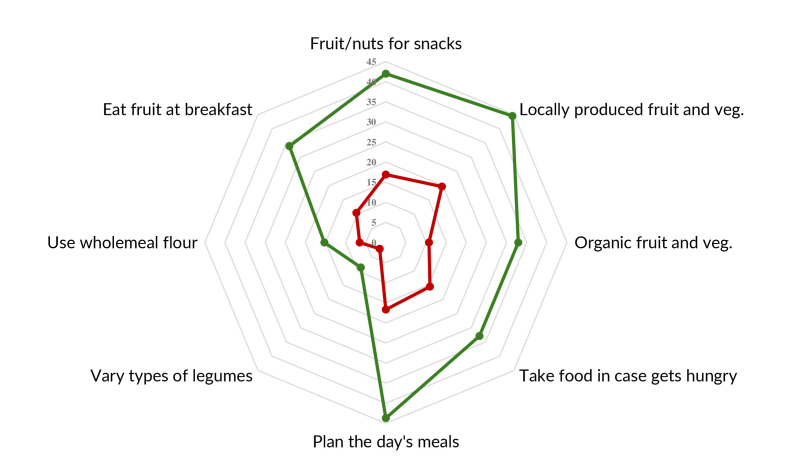
Prevalence of the planning items of the adherence scale for dietary practices recommended by the Dietary Guidelines, according to their dimensions, among people with obesity taking part in the Health Fitness Program. Belo Horizonte, 2022-2023 (n=1,109)

**3B:**
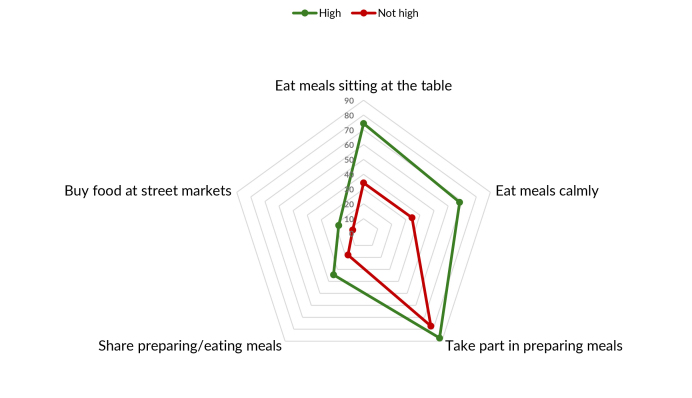
Prevalence of the domestic organization items of the adherence scale for dietary practices recommended by the Dietary Guidelines, according to their dimensions, among people with obesity taking part in the Health Fitness Program. Belo Horizonte, 2022-2023 (n=1,109)

**3C:**
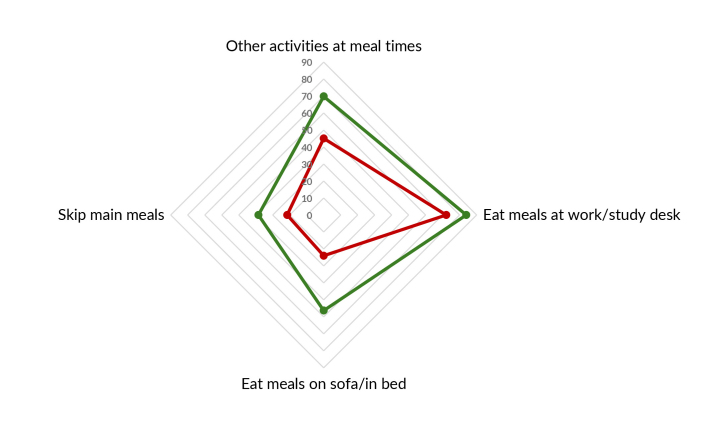
Prevalence of the ways of eating items of the adherence scale for dietary practices not recommended by the Dietary Guidelines, according to their dimensions, among people with obesity taking part in the Health Fitness Program. Belo Horizonte, 2022-2023 (n=1,109)

**3D:**
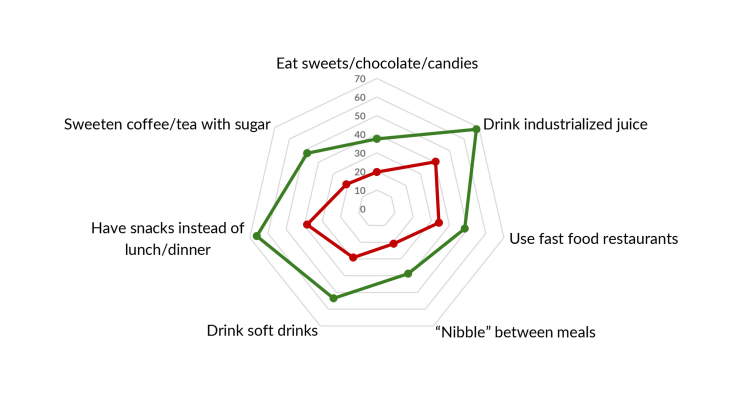
Prevalence of the ways of eating items of the adherence scale for dietary practices not recommended** by the Dietary Guidelines, according to their dimensions, among people with obesity taking part in the Health Fitness Program. Belo Horizonte, 2022-2023 (n=1,109)

In the multivariate model adjusted for sex, we found that elderly individuals were 47.0% more likely to have high adherence to the Dietary Guidelines (OR 1.47; 95%CI 1.1; 2.0). On the other hand, those in the first income tertile had lower odds of adherence (OR 0.58; 95%CI 0.41; 0.81), as did those who self-rated their health as regular (OR 0.73; 95%CI 0 55; 0.96) and poor/very poor (OR 0.26; 95%CI 0.13; 0.52) (Table 2).

**Table 2:** Sex-adjusted association between sociodemographic and health characteristics, and high adherence to dietary practices recommended by the Dietary Guidelines, among people with obesity taking part in the Health Fitness Program. Belo Horizonte, 2022-2023 (n=1,109)

Variables	OR (95%CI)	p-value
**Stage of life**		
Adult	Reference	-
Elderly	1.47 (1.10; 2.00)	0.013
**Professional occupation**		
Housewife	Reference	-
Retired	1.05 (0.75; 1.48)	0.773
Unemployed	0.67 (0.33; 1.34)	0.252
Other	0.93 (0.66; -1.44)	0.648
**Monthly per capita income**		
1st tertile (BRL 57-606)	Reference	-
2nd tertile (BRL 625-BRL 1,212)	0.75 (0.54; 1.04)	0.085
3rd tertile (BRL 1,250-BRL 10,000)	0.58 (0.41; 0.81)	0.002
Hypertension		
No	Reference	-
Yes	0.99 (0.75; 1.30)	0.094
**Type 2 diabetes**		
No	Reference	-
Yes	1.31 (0.99; 1.75)	0.062
**Self-rated health**		
Good/very good	Reference	-
Regular	0.73 (0.55; 0.96)	<0.001
Poor/very poor	0.26 (0.13; 0.52)	0.013
**Length of time in the Health Fitness Program**		
1st tertile (0-2.8)	Reference	-
2nd tertile (2.9-4.6)	0.87 (0.64; 1.18)	0.359
3rd tertile (4.7-16.5)	0.80 (0.59; 1.12)	0.210
		

## Discussion

Almost half of the Health Fitness Program participants with obesity showed high adherence to the dietary practices recommended by the Dietary Guidelines, with the odds being higher among elderly individuals, with higher income and better self-rated health. The most prevalent eating practices aligned with the Dietary Guidelines recommendations were eating while sitting at the table and taking part in meal preparation; and among the least prevalent, replacing beans with other legumes, using whole wheat flour and the habit of eating breakfast.

Prevalence of high adherence to the Dietary Guidelines recommendations found by us was higher than the prevalence found by (23%) by Gabe & Jaime (14), when investigating a convenience sample of Brazilian macro-regions. This difference may be related to the participants in this study taking part in a health promotion service, which aims to promote healthy lifestyles, including adequate and healthy eating (16).

Despite the positive results, the Dietary Guidelines recommendations need to be intensified among individuals with low and medium adherence. When considering that adherence to appropriate and healthy eating practices is influenced by individual and environmental factors, it is crucial that obstacles to their being adopted, and the possible means to overcome them (1) are addressed through structural interventions and food and nutrition education. As such, analysis of the dimensions of the scale can contribute to identifying priorities for intervention. In this study, the planning dimension was the weakest among individuals with low adherence to the Dietary Guidelines, highlighting the importance of working on aspects related to buying food, the weekly menu and sharing household activities, which can even contribute to reducing the effects of lack of time, this being one of the main obstacles highlighted by the Dietary Guidelines (1). It is also necessary to work on the relevance of involvement with meals eaten at home, eating breakfast and sitting at the table (1). 

Additionally, we identified reduced use of wholemeal flour, buying food at open-air markets and diversifying intaken of legumes beyond beans alone. Both aspects may be related to participants having lower income and the local food culture, which does not favor such eating habits. However, the importance of consuming other foods rich in fiber, such as fruit and vegetables, and continuing to consume beans as the main component of the legume group can be reinforced, with the aim of promoting an adequate and healthy diet.

High adherence to the Dietary Guidelines recommendations was associated with stage of life, as also found by Gabe & Jaime (14). This relationship may be linked to the ways of living taken on as age increases and also greater prevalence of diseases that require greater care with nutrition and health (17-19). Furthermore, elderly individuals, in general, have more time available to dedicate to buying/preparing food and using their culinary skills (19).

Another factor we found to be associated with high adherence to the Dietary Guidelines recommendations was family income, this being an important determinant of access to adequate and healthy food (20-21). In this study, we found that users in the first income tertile had lower odds of high adherence to the Dietary Guidelines recommendations. Considering that the areas surrounding the Health Fitness Program units in Belo Horizonte are characterized by lower density of establishments that sell healthy foods, limited purchasing power and access limitations can be additional difficulties to adherence (22-23). Higher income is associated with greater food purchasing power, especially given the increasing cost of unprocessed and minimally processed foods in Brazil (24). Furthermore, it contributes to overcoming the limited supply of these foods in local commerce, by facilitating physical access to foods with better quality and price in other regions (25).

High adherence to the Dietary Guidelines recommendations was also greater among participants with good/very good self-rated health. As it is a health promotion service, participation in the Program may result in adoption of healthier behaviors and greater readiness to do so, resulting in more positive self-rated health and, consequently, people seeking to make changes in their diet (26).

Despite its results, this study has limitations that must be considered. Conducting it in a Primary Health Care promotion service, despite being a potential, may also limit its results being inferred in relation to the general population. Program users may be more exposed to dietary guidance provided by nutritionists or other health professionals, in addition to mostly being women (26). Such aspects may have an impact on adherence to the Dietary Guidelines considering that women and individuals more exposed to health guidelines tend to adopt healthier behaviors when compared to their counterparts. However, 76% of the Brazilian population is covered by Primary Health Care (27), and Brazil currently has 3,437 Health Fitness Program units (28). Another possible limitation was the assessment of adherence based on self-rating (Likert scale), which may be subject to information and social desirability bias. In order to minimize these biases, we used a scale validated for the Brazilian population (13).

Notwithstanding, it is noteworthy that this is the first study to analyze the degree of adherence to practices recommended by the Dietary Guidelines among SUS users with obesity, generating important contributions to the body of evidence. The use of a validated tool, which measures different dimensions of adequate and healthy eating, in a representative sample of a public health service, is equally innovative, allowing a comprehensive assessment of the eating practices of participants in an important Brazilian Primary Health Care service. Therefore, the potential of this study to contribute to the improvement of actions to implement the Dietary Guidelines in Primary Health Care stands out, especially among people with obesity.

In conclusion, stage of life, better income and positive self-rated health were factors associated with high adherence to dietary practices recommended by the Dietary Guidelines among people with obesity participating in the Health Fitness Program in Belo Horizonte. These results highlight the need to target efforts towards the development of actions to promote adequate and healthy eating, based on the Dietary Guidelines, among adults and those belonging to vulnerable groups.

## References

[1] Ministério da Saúde (BR) (2014). Guia alimentar para a população brasileira.

[2] Ministério da Saúde (BR) (2018). Política Nacional de Promoção da Saúde. Anexo I da Portaria de
Consolidação nº 2, de 28 de setembro de 2017, que consolida as normas sobre
as políticas nacionais de saúde do SUS.

[3] Ministério da Saúde (BR) (2012). Política Nacional de Alimentação e Nutrição.

[4] Gabe KT, Jaime PC (2019). Development and testing of a scale to evaluate diet according to
the recommendations of the Dietary Guidelines for the Brazilian
Population. Public Health Nutrition.

[5] Magalhães ACO, Marques CG, Lucin GA, Nakamoto FP, Tufik S, Thomatieli-Santos RV (2024). The relationship between sleep- and circadian rhythm-related
parameters with dietary practices and food intake of sedentary adults: a
cross-sectional study. Sleep Biol.

[6] Gabe KT, Costa CS, Dos Santos FS, Souza TN, Jaime PC (2023). Is the adherence to the food practices recommended by the dietary
guidelines for the Brazilian population associated with diet
quality?. Appetite.

[7] Ministério da Saúde (BR) (2021). Instrutivo de Abordagem Coletiva para o Manejo da Obesidade no Sistema
Único de Saúde.

[8] Ministério da Saúde (BR) (2020). Protocolo Clínico e Diretrizes Terapêuticas do Sobrepeso e Obesidade em
Adultos.

[9] Freitas PP, Lopes MS, Araujo JR, Cunha RB, Duarte CK, Lopes ACS (2024). Protocol of Randomized Controlled Community Trial (RCCT) for
obesity management in Brazilian primary health care. BMC Health Services Research.

[10] Alves JA, Gibson CL (2019). States and Capitals of Health: Multilevel Health Governance in
Brazil. Latin American Politics and Society.

[11] Ministério da Saúde (Brasil) (2014). Cartilha Informativa: Academia da Saúde.

[12] Prefeitura Municipal de Belo Horizonte (2024). Academia da Cidade.

[13] Gabe KT, Jaime PC (2022). Convergent validity and invariance analysis of a scale to measure
adherence to eating practices recommended by the Dietary Guidelines for the
Brazilian Population. Rev Bras Epidemiol.

[14] Santos TSSS, Carvalho MCR, Duarte CK, Jaime PC, Lopes ACS (2023). Medida do equilíbrio de decisões para redução do peso corporal
entre pessoas com sobrepeso ou obesidade: uma revisão
sistemática. DEMETRA: Alimentação, Nutrição & Saude.

[15] Gabe KT, Costa CS, Santos FS, Souza TN, Jaime PC (2018). Práticas alimentares segundo o Guia alimentar para a população
brasileira: fatores associados entre brasileiros adultos,
2018. Epidemiol Serv Saude.

[16] Ministério da Saúde (BR) (2019). Monitoramento do Programa Academia da Saúde: Ciclo 2019.

[17] Martins AL da C, Bertin RL, Calao KMFN, Medeiros CO (2020). Habilidades culinárias de idosos praticantes de atividades
físicas aquáticas. RSD.

[18] Silva SRDA, Araújo KMST, Xavier MLA, Silva ALO (2024). Representações Sociais Da Alimentação Saudável Para Pessoa Idosa:
Uma Revisão Integrativa: Representações Sociais Da Alimentação Saudável Para
Pessoa Idosa. Rev Enferm.

[19] Mills S, White M, Brown H, Wrieden W, Kwasnicka D, Halligan J (2017). Health and social determinants and outcomes of home cooking: A
systematic review of observational studies. Appetite.

[20] Santin F, Gabe KT, Levy RB, Jaime PC (2013). Food consumption markers and associated factors in Brazil:
distribution and evolution, Brazilian National Health Survey, 2013 and
2019. Cad Saúde Publica.

[21] Oliveira N, Santin F, Paraizo TR, Sampaio JP, Moura Nunes, Canella DS (2021). Baixa variedade na disponibilidade domiciliar de frutas e
hortaliças no Brasil: Dados das POF 2008-2009 e 2017-2018. Cienc Saude Coletiva.

[22] Lopes MS, Martiniano MO, De Freitas PP, Carvalho MCR, Sales DM, Lopes ACS (2022). Comércio de alimentos para consumo imediato no entorno do
Programa Academia da Saúde: uma análise segundo
desigualdades. Cienc. saude coletiva.

[23] Menezes MC, Costa BVL, Oliveira CL, Lopes ACS (2017). Local food environment and fruit and vegetable consumption: An
ecological study. Preventive Medicine Reports.

[24] Andrade GC, Caldeira TCM, Mais LA, Bortoletto AP, Claro RM (2024). Food price trends during the COVID-19 pandemic in
Brazil. PLoS One.

[25] Marquezine TC, Vandevijvere S, Swinburn B, Claro RM (2024). Differences in the cost and environmental impact between the
current diet in Brazil and healthy and sustainable diets: a modeling
study. Nutrition Journal.

[26] Mendonça RD, Lopes MS, Carvalho MCR, De Freiras PP, Lopes ACS (2020). Adherence to healthy lifestyle in the Programa Academia da
Saúde. Revista Brasileira de Atividade Física e Saúde.

[27] Instituto de Estudos para Políticas de Saúde (IEPS) (2021). Cobertura da Atenção Básica, 2021.

[28] Ministério da Saúde (BR) (2022). DATASUS. Tabnet.

