# Supplementary material for: Adherence to dietary practices recommended by the Dietary Guidelines for the Brazilian Population among people with obesity: baseline of a community trial carried out at the Health Fitness Program in Belo Horizonte, Brazil, 2022-2023
Source: Epidemiol Serv Saude. 2025 Apr 28;34:e20240287. doi: 10.1590/S2237-96222024v34e20240287.en (PMC12039795; doi:10.1590/S2237-96222024v34e20240287.en)
Supplement: Translation

# Translation: Adesão às práticas alimentares recomendadas pelo Guia Alimentar para a População Brasileira entre pessoas com obesidade: linha de base de ensaio comunitário realizado no Programa Academia da Saúde de Belo Horizonte, 2022-2023

**DOI:** 10.1590/S2237-96222024v34e20240287.pt

**Authors:** Mariana Souza Lopes, Vitória Marli Serafim Moreira Silva, Nathália Luíza Ferreira, Maria Cecília Ramos de Carvalho, Patrícia Pinheiro de Freitas, Aline Cristine Souza Lopes

**Affiliations:** 1 Universidade Federal da Paraíba, João Pessoa, Paraíba, Brasil; 2 Universidade Federal de Minas Gerais, Belo Horizonte, Minas Gerais, Brasil; 3 Universidade Federal de Lavras, Lavras, Minas Gerais, Brasil; 4 Universidade Federal dos Vales do Jequitinhonha e Mucuri, Diamantina, Minas Gerais, Brasil

**Keywords:** Estudos Observacionais, Guias Alimentares, Obesidade, Fatores Sociodemográficos, Atenção Primária à Saúde

## Abstract

**Objetivo:**

Analisar os fatores sociodemográficos e de saúde associados à alta adesão às práticas alimentares recomendadas pelo *Guia Alimentar para População Brasileira* entre usuários com obesidade do Programa Academia da Saúde de Belo Horizonte, Minas Gerais.

**Métodos:**

Estudo transversal em amostra representativa do Programa Academia da Saúde com participantes com obesidade e idade ≥20 anos. Coletou-se dados sociodemográficos e de saúde, e aplicou a escala de adesão ao Guia Alimentar, classificada em: baixa (<32 pontos), média (32-41 pontos) e alta (>41 pontos). Realizou-se regressão logística multivariada.

**Resultados:**

Participaram 1.109 indivíduos, sendo a maioria mulheres (92,5%) e com alguma doença crônica (85,8%). Quanto às condições de saúde, a maioria avaliou sua saúde como muito boa/boa (62,2%), 62,8% possuíam Hipertensão e 68,0% obesidade I. Alta adesão ao Guia Alimentar foi de 46,0%. Após ajuste, a alta adesão foi associada a ser idoso [razão de chances (*odds ratio*, OR 1,47; IC95% 1,1; 2,0), ter maior renda (OR 0,58; IC95% 0,41; 0,81) e melhor percepção de saúde (regular: OR 0,73; IC95% 0,55; 0,96; ruim/muito ruim: OR 0,26; IC95% 0,13; 0,52).

**Conclusão:**

A alta adesão ao Guia foi associada à fase da vida, melhor renda e autoavaliação positiva da saúde, evidenciando a necessidade de direcionar esforços para adultos e grupos vulneráveis.

## Introdução

Os princípios e as recomendações para alimentação adequada e saudável dos brasileiros são apresentados no *Guia Alimentar para a População Brasileira*, documento reconhecido internacionalmente. O Guia tem como pressupostos o direito à saúde e à alimentação adequada, e os múltiplos determinantes das práticas alimentares (1). Seus princípios consideram os alimentos e suas combinações, bem como as dimensões culturais e sociais das práticas alimentares. Além disso, ponderam a interdependência entre alimentação e os sistemas alimentares, valorizando os diferentes saberes para a formulação de recomendações alimentares (1).

Para nortear as recomendações para uma alimentação adequada e saudável, o Guia preconiza a regra de ouro “Prefira sempre alimentos in natura ou minimamente processados e preparações culinárias a alimentos ultraprocessados”, além de considerar os modos de comer e a comensalidade (1). Essa perspectiva é inovadora e avança na concretização das políticas de saúde, alimentação e nutrição (2,3).

Para apoiar a implementação do Guia no Sistema Único de Saúde (SUS) diferentes materiais vêm sendo desenvolvidos. Entre esses, destaca-se o teste “Como está sua alimentação”, que avalia, de modo multidimensional e qualitativo, a adesão às recomendações do Guia (4). Estudos conduzidos com indivíduos adultos e idosos identificaram associação significativa entre características sociodemográficas e adesão ao Guia (5,6). Entretanto, não exploraram essa adesão entre indivíduos com obesidade e usuários da Atenção Primária à Saúde (APS).

A APS é um cenário privilegiado para a implementação do Guia por abarcar ações de promoção, manutenção e recuperação da saúde, além de sua proximidade com a comunidade e alcance populacional (7). Entretanto, quando se trata de grupos específicos, como pessoas com obesidade, preconiza-se que as diretrizes do Guia sejam adaptadas. 

Segundo o Protocolo Clínico e Diretrizes Terapêuticas de Sobrepeso e Obesidade em Adultos, o cuidado de pessoas com obesidade deve contemplar a promoção de escolhas alimentares adequadas e saudáveis (8). Mas, para isso, é necessário investigar as práticas alimentares e propor ações que vão além do consumo de grupos de alimentos e nutrientes, abarcando os modos de comer e a comensalidade, entre outras práticas alimentares (1). Nesse sentido, este artigo, objetivou analisar os fatores sociodemográficos e de saúde associados à alta adesão às práticas alimentares recomendadas pelo Guia entre usuários com obesidade do Programa Academia da Saúde de Belo Horizonte, Minas Gerais.

## Métodos

### Delineamento do estudo 

Trata-se de estudo transversal, de Triagem e Linha de Base de dois Ensaios Comunitários Controlados Randomizados (ECCR) conduzidos com usuários com obesidade do Programa Academia da Saúde de Belo Horizonte, Minas Gerais, durante o período de maio de 2022 a junho de 2023, sendo as etapas de Triagem e de Linha de Base realizadas de maio a outubro de 2022. Os ECCR foram realizados simultaneamente visando avaliar a efetividade de intervenções nutricionais coletivas para manejo da obesidade entre usuários do Programa Academia da Saúde, segundo a gravidade da condição de obesidade (9).

### Contexto

Belo Horizonte é a capital do estado de Minas Gerais e conta com aproximadamente 2.3 milhões de habitantes, sendo a sexta cidade mais populosa do Brasil e referência nacional na organização da APS (10).

O Programa Academia da Saúde é um serviço de promoção da saúde da APS brasileira, desde 2011, ofertando práticas corporais/atividade física; ações de promoção da alimentação adequada e saudável, de mobilização da comunidade, de modos de vida saudáveis, entre outras (11). Em Belo Horizonte, o serviço oferta prática regular de exercícios físicos, orientada por profissionais de Educação Física, para usuários com idade ≥18 anos ingressantes no serviço por demanda espontânea ou referenciados pela rede de saúde. Atualmente, são mais de 80 unidades no município, distribuídas nas nove regionais administrativas, atendendo a cerca de 15.000 usuários (12).

### Tamanho do estudo

Realizou-se processo amostral unificado para os ECCR mediante amostragem aleatória simples estratificada pelas nove regionais do município. O plano amostral foi conduzido por estatístico externo, sem envolvimento das pesquisadoras. Foram consideradas elegíveis as unidades do Programa Academia da Saúde com funcionamento matutino (principal turno), que não haviam realizado intervenções nutricionais para manejo da obesidade nos últimos dois anos e que possuíam profissional de Educação Física durante a pesquisa (9).

Para o cálculo amostral, considerou-se a prevalência de obesidade grau I de 20,0% (Índice de Massa Corporal – IMC 30-35kg/m²), diferença do peso corporal dos participantes entre os grupos de alocação da intervenção de 5,0%, e acréscimo de 35,0% para eventuais perdas, estimadas a partir de estudos anteriores. A amostra final constou de 5.652 indivíduos distribuídos em 59 unidades do Programa Academia da Saúde, sendo representativa do serviço de saúde no município, com 95,0% de confiança e erro <2,8% (9).

### Participantes

Nas unidades do Programa Academia da Saúde selecionadas (n=59) foram convidados para participar da Triagem todos os usuários com ≥20 anos de idade frequentes às atividades do Programa Academia da Saúde (participação em ao menos uma aula no mês anterior), excluindo-se as mulheres gestantes e indivíduos com dificuldade cognitiva que impedisse de responder ao questionário (9).

Após participar da Triagem, os indivíduos que apresentavam obesidade (IMC≥30kg/m²); que tinham desejo e disponibilidade para participar de grupos com duração de seis meses ou mais; e prontidão de mudança para redução do peso, segundo estágios de mudança de “preparação com alta autoeficácia”, “ação” ou “manutenção” propostos pelo Modelo Transteórico (7), foram elegíveis para participar da próxima etapa do estudo, a Linha de Base (n=1.427). Na Linha de Base todos aqueles que responderam ao teste “Como está sua alimentação?” (9), variável resposta deste estudo, foram incluídos. Dezoito participantes não responderam completamente o teste e foram excluídos. Portanto, após aplicação dos critérios de inclusão e de exclusão deste estudo, a amostra final foi composta por 1.109 indivíduos com obesidade, interesse/disponibilidade para participar das etapas seguintes da pesquisa, com prontidão para redução do peso corporal e que responderam ao teste “Como está sua alimentação?”.

### Fontes de dados/medidas 

A coleta de dados foi realizada em duas etapas: Triagem e Linha de Base. Na Triagem, realizou-se avaliação antropométrica e investigou-se variáveis sociodemográficas, desejo e tempo de participar de grupos, prontidão de mudança para redução do peso, morbidade referida, percepção de saúde e participação no Programa Academia da Saúde. Na Linha de Base, realizada na sequência imediata da Triagem, aplicou-se o teste “Como está sua alimentação?” validado para a população brasileira e com confiabilidade (coeficiente de correlação intraclasse 0,82) (4).

O peso corporal (kg) foi aferido em tomada única por balança digital com capacidade máxima de 200kg; e a altura (cm) avaliada com auxílio de estadiômetro portátil, com escala de 0,35m até 2,13m. Ambas as medidas foram realizadas na etapa da Triagem (9).

Os instrumentos empregados foram baseados em inquéritos nacionais e internacionais, na experiência pregressa do grupo de pesquisa e em questionários validados. As entrevistas foram realizadas face a face, com o uso de tablets. Os dados foram coletados no aplicativo gratuito Epicollect5 e submetidos a análise de consistência. Em caso de instabilidade do aplicativo, a coleta era realizada em questionário impresso, com posterior digitação e análise de consistência (9).

A coleta dos dados foi realizada no horário de funcionamento do Programa Academia da Saúde e as entrevistas agendadas conforme disponibilidade dos usuários. A equipe de entrevistadores incluiu profissionais e graduandos da área da saúde, supervisionados por nutricionistas e supervisora geral, com apoio das coordenadoras da pesquisa (9).

### Variáveis

### Variável desfecho: adesão às práticas alimentares recomendadas pelo Guia Alimentar

Para mensurar a adesão ao Guia, utilizou-se escala multidimensional de 24 itens, a qual foi avaliada previamente por análises de validação convergente e de invariância para subgrupos de sexo, idade e escolaridade e resultados adequados (13). A escala compreende quatro dimensões: planejamento, organização doméstica, modos de comer e escolha dos alimentos. Cada item é seguido de escala Likert com as opções de resposta: discordo fortemente; discordo; concordo; concordo fortemente. Para cada item são atribuídos valores de 0 a 3, sendo o escore computado pela soma das respostas, podendo variar de 0 a 72. Os 13 primeiros itens da escala são alinhados às recomendações do Guia e pontuados de modo que a resposta “concordo fortemente” receba 3 pontos. E os 11 itens finais são opostos às suas recomendações, sendo pontuados de maneira inversa, com a resposta “discordo fortemente” recebendo 3 pontos (13).

Para classificação da adesão às recomendações, utilizou-se os seguintes pontos de corte: baixa (até 31 pontos), média (32-41 pontos) e alta adesão (>41 pontos) (15). Na análise multivariada, as categorias “baixa adesão” e “média adesão” foram agrupadas.

### Variáveis explicativas de interesse principal

As variáveis sociodemográficas investigadas foram: sexo (masculino; feminino), fase da vida (adulto: 20-59 anos; idoso: ≥60 anos), escolaridade (anos), tercil de renda *per capita* mensal e situação conjugal (com; sem companheiro). A renda foi obtida pela renda familiar total dividida pelo número de moradores do domicílio. 

Em relação à saúde, investigou-se: percepção de saúde (muito boa/boa; razoável; ruim/muito ruim), tempo de participação no Programa Academia da Saúde (anos) e morbidades referidas (doença cardiovascular, dislipidemia, apneia do sono, doenças articulares, diabetes mellitus tipo II - DM tipo II, hipertensão arterial - HA, doença hepática e doença mental). As prevalências de HA e DM tipo II (sim; não) foram apresentadas separadamente, e em conjunto com as demais comorbidades segundo o número de doenças crônicas (0; 1; 2; 3 ou mais). O tempo de participação no Programa foi obtido pela subtração da data de entrevista da Triagem pela data de ingresso, obtida nos registros do serviço de saúde. 

Para diagnóstico de obesidade, utilizou-se o IMC (peso/altura²), categorizado segundo a Organização Mundial da Saúde. Já a gravidade da obesidade (sim; não) foi identificada mediante adaptação dos critérios da Portaria 424/2013 para realização de cirurgia bariátrica, sendo considerada obesidade grave: IMC ≥40 Kg/m²; e IMC >35 kg/m² com DM tipo II e/ou HA, apneia do sono ou doenças articulares (7).

A prontidão para o tratamento da obesidade foi analisada conforme a Estratificação de Grupos para Manejo da Obesidade proposta no *Instrutivo de Abordagem Coletiva para o Manejo da Obesidade no SUS* (7). Essa Estratificação propõe que a prontidão de mudança para o tratamento da obesidade seja avaliada pela disponibilidade e desejo de participar de grupos, e pelos estágios de mudança de comportamento e a autoeficácia para redução do peso, segundo o Modelo Transteórico. Os estágios de mudança foram avaliados a partir de algoritmo proposto pelo Ministério da Saúde, contendo respostas reciprocamente excludentes visando identificar a intenção do indivíduo em iniciar mudanças para reduzir seu peso e o tempo decorrido desde o início das mudanças. A autoeficácia consiste na confiança que o indivíduo tem para realizar e manter a mudança de comportamento, sendo mensurada por escala validada contendo três questões com opções de resposta “nada confiante”; “pouco confiante”; “moderadamente confiante”; “muito confiante”; “completamente confiante”. Considerou-se como autoeficácia elevada a presença de, pelo menos, duas questões avaliadas como muito confiante ou completamente confiante (15,7).

Foram considerados como indivíduos com prontidão de mudança para redução do peso aqueles nos estágios de “preparação com autoeficácia elevada”, “ação” ou “manutenção” (7). 

### Viés

Para garantia do controle e da qualidade dos dados, e adequada validade interna do estudo, toda a equipe foi treinada previamente para a coleta de dados, sob a supervisão de um profissional de saúde, além de contar com manuais e logística de campo para orientar as atividades (9).

### Métodos estatísticos

Para verificar a associação entre as características dos participantes e o grau de adesão às práticas alimentares recomendadas pelo Guia, utilizou-se os testes estatísticos Qui-quadrado de Pearson para variáveis categóricas e Kruskal-Wallis para as numéricas. 

Para avaliar a relação entre a alta adesão ao Guia (sim; não) e as características sociodemográficas e de saúde dos participantes, utilizou-se regressão logística multivariada, com estimativas de razão de chances (*odds ratio*, OR) e respectivos intervalos de confiança de 95,0% (IC95%). O modelo foi ajustado por sexo.

As análises foram realizadas no STATA/SE, com nível de significância de 5,0%.

## Resultados

Dos 1.109 indivíduos elegíveis ao estudo, a maioria eram mulheres (92,5%), idosos (56,1%) e com mais de três anos de participação no Programa Academia da Saúde (66,5%). Quanto às condições de saúde, a maioria avaliou sua saúde como muito boa/boa (62,2%), 62,8% possuíam HA e 68,0% obesidade I (Tabela 1).

**Tabela 1:** Características sociodemográficas, de saúde e estado nutricional segundo grau de adesão às práticas alimentares recomendadas pelo Guia Alimentar entre usuários com obesidade do Programa Academia da Saúde. Belo Horizonte, 2022-2023 (n=1.109)

Variáveis	Amostra total n=1.109 (100%)	Adesão ao *Guia Alimentar para a População Brasileira*			p-valor
		Alta n=510	Média n=418	Baixa n=181	
Sexo					0,404^a^
Masculino	7,5	7,3	8,6	5,5	
Feminino	92,5	92,7	91,4	94,5	
**Fase da vida**					<0,001^a^
Adulto	43,9	35,5	45,5	64,1	
Idoso	56,1	64,5	54,5	35,9	
**Situação conjugal**					0,671^a^
Com companheiro	56,0	56,9	54,3	57,5	
Sem companheiro	44,0	43,1	45,7	42,5	
**Escolaridade^c^; mediana** (P_25_-P_75_)^f^	8,0 (4,0-11,0)	9,0 (4,0-11,0)	8,0 (4,0-11,0)	8,0 (4,0-11,0)	0,298^b^
**Renda per capita mensal em reais** (tercis)					<0,001^a^
1º tercil (R$57,00-606,00)	36,0	29,4	39,5	46,4	
2º tercil (R$625,00-R$1.212,00)	38,0	39,4	36,1	38,7	
3º tercil (R$1.250,00-R$10.000,00)	26,0	31,2	24,4	14,9	
**Ocupação profissional**					<0,001^a^
Do lar	30,2	28,8	30,4	33,7	
Aposentado/a	36,2	41,8	34,9	23,2	
Desempregado/a	4,3	2,6	5,0	7,7	
Outro	29,3	26,9	29,7	35,4	
**Percepção de saúde** ^d^					<0,001^a^
Boa/Muito boa	62,2	68,0	58,6	54,1	
Regular	32,4	29,6	34,6	35,4	
Ruim/muito ruim	5,4	2,4	6,7	10,5	
Hipertensão arterial	62,8	64,8	63,9	54,7	0,045^a^
Diabetes mellitus tipo II	29,9	32,7	29,7	22,2	0,030^a^
**Número de doenças crônicas; mediana** (P_25_-P_75_)^f^	2,0 (1,0-3,0)	2,0 (1,0-3,0)	2,0 (1,0-3,0)	2,0 (1,0-3,0)	0,325^b^
Nenhuma	14,2	12,3	15,3	17,1	0,366^a^
1	25,2	26,5	23,7	24,9	
2	25,7	25,9	24,2	28,7	
>3	34,9	35,3	36,8	29,3	
**Grau de obesidade**					0,083^a^
I	68,0	72,1	64,0	66,0	
II	24,8	22,2	27,0	26,8	
III	7,2	5,7	9,0	7,2	
**Gravidade da obesidade**					0,230^a^
Não grave	86,0	87,5	85,9	82,3	
Grave	14,0	12,5	14,1	17,7	
**Prontidão para tratar obesidade**					0,496^a^
Sim	62,2	65,5	62,2	58,6	
Não	37,8	36,5	37,8	41,4	
**Tempo de participação no Programa Academia da Saúde** (anos)^e^					0,003^a^
1º tercil (0-2,8)	33,5	29,2	34,4	43,4	
2º tercil (2,9-4,6)	33,2	33,3	32,9	33,5	
3º tercil (4,7-16,5)	33,3	37,5	32,7	23,1	

^a^Teste Qui-Quadrado de Pearson; ^b^Teste Kruskal-Wallis; ^c^4 dados faltantes; ^d^2 dados faltantes; ^e^49 dados faltantes; ^f^P25=Percentil 25; P75=Percentil 75.

O escore final da escala de adesão ao Guia variou de 14 a 66 pontos, com mediana de 41 pontos (dados não apresentados em tabela), sendo a prevalência de alta adesão de 46,0%, de média adesão de 37,7% e de baixa adesão de 16,3% (dados não apresentados).

A Figura 1 apresenta as práticas alimentares alinhadas ao Guia, e a Figura 2, aquelas não alinhadas. A maior prevalência de prática positiva foi identificada para participar do preparo dos alimentos em casa 91,1% (81,9% - concordo fortemente + 9,2% - concordo) (Figura 1), e a negativa foi beber café ou chá com açúcar (53,4%) (Figura 2).

**Figura 1:** Prevalências dos itens da escala de adesão às práticas alimentares alinhadas às recomendações do Guia Alimentar entre usuários com obesidade do Programa Academia da Saúde. Belo Horizonte, 2022-2023 (n=1.109)

**Figura 2:** Prevalências dos itens da escala de adesão às práticas alimentares não alinhadas às recomendações do Guia Alimentar entre usuários com obesidade do Programa Academia da Saúde. Belo Horizonte, 2022-2023 (n=1.109)

As características sociodemográficas e de saúde associadas a alta adesão às recomendações do Guia foram: fase da vida, renda, percepção de saúde, relato de HAS e DM tipo II e tempo de participação no Programa Academia da Saúde (p-valor<0,050) (Tabela 1).

A distribuição dos itens da escala segundo a alta adesão ao Guia (sim; não) (Figura 3) mostrou baixas prevalências em ambos os grupos para “uso de farinha integral” (15,3% e 6,5%, respectivamente), “compra de alimentos nas feiras” (17,6% e 7,7%, nesta ordem) e “substituição de feijão por outras leguminosas” (8,8% e 2,2%, respectivamente); e maior diferença entre os grupos em relação à realização das “refeições sentado/a à mesa” (74,3% e 34,2%, nessa ordem). Quanto às dimensões da escala, identificou-se maiores diferenças para o planejamento da alimentação (3A), e menores para os modos de comer (3C). 

**Figura 3:** Prevalências dos itens da escala de adesão às práticas alimentares recomendadas* e não recomendadas** pelo Guia Alimentar entre usuários com obesidade do Programa Academia da Saúde segundo suas dimensões. Belo Horizonte, 2022-2023 (n=1.109)

**3A:** Prevalências dos itens de planejamento da escala de adesão às práticas alimentares recomendadas pelo Guia Alimentar entre usuários com obesidade do Programa Academia da Saúde segundo suas dimensões. Belo Horizonte, 2022-2023 (n=1.109)

**3B:** Prevalências dos itens de organização doméstica da escala de adesão às práticas alimentares recomendadas pelo Guia Alimentar entre usuários com obesidade do Programa Academia da Saúde segundo suas dimensões. Belo Horizonte, 2022-2023 (n=1.109)

**3C:** Prevalências dos itens de modos de comer da escala de adesão às práticas alimentares não recomendadas pelo Guia Alimentar entre usuários com obesidade do Programa Academia da Saúde segundo suas dimensões. Belo Horizonte, 2022-2023 (n=1.109)

**3D:** Prevalências dos itens de modos de comer da escala de adesão às práticas alimentares não recomendadas pelo Guia Alimentar entre usuários com obesidade do Programa Academia da Saúde segundo suas dimensões. Belo Horizonte, 2022-2023 (n=1.109)

No modelo multivariado ajustado por sexo, verificou-se que indivíduos idosos tiveram 47,0% mais chances de apresentar alta adesão ao Guia (OR 1,47; IC95% 1,1; 2,0). Por outro lado, aqueles no primeiro tercil de renda tiveram menores chances (OR 0,58; IC95% 0,41; 0,81), assim como aqueles que avaliaram sua saúde como regular (OR 0,73; IC95% 0,55; 0,96) e ruim/muito ruim (OR 0,26; IC95% 0,13; 0,52) (Tabela 2).

**Tabela 2:** Associação ajustada por sexo entre características sociodemográficas e de saúde, e alta adesão às práticas alimentares recomendadas pelo Guia Alimentar entre usuários com obesidade do Programa Academia da Saúde. Belo Horizonte, 2022-2023 (n=1.109)

Variáveis	OR (IC95%)	p-valor
**Fase da vida**		
Adulto	Referência	-
Idoso	1,47 (1,10; 2,00)	0,013
**Ocupação profissional**		
Do lar	Referência	-
Aposentado	1,05 (0,75; 1,48)	0,773
Desempregado	0,67 (0,33; 1,34)	0,252
Outro	0,93 (0,66; -1,44)	0,648
**Renda per capita mensal**		
1º tercil (R$57,00-606,00)	Referência	-
2º tercil (R$625,00-R$1.212,00)	0,75 (0,54; 1,04)	0,085
3º tercil (R$1.250,00-R$10.000,00)	0,58 (0,41; 0,81)	0,002
**Hipertensão arterial**		
Não	Referência	-
Sim	0,99 (0,75; 1,30)	0,094
**Diabetes tipo II**		
Não	Referência	-
Sim	1,31 (0,99; 1,75)	0,062
**Percepção de saúde**		
Boa/muito boa	Referência	-
Regular	0,73 (0,55; 0,96)	<0,001
Ruim/muito ruim	0,26 (0,13; 0,52)	0,013
**Tempo de participação no Programa Academia da Saúde**		
1º tercil (0-2,8)	Referência	-
2º tercil (2,9-4,6)	0,87 (0,64; 1,18)	0,359
3º tercil (4,7-16,5)	0,80 (0,59; 1,12)	0,210
		

## Discussão

Quase metade dos usuários do Programa Academia da Saúde com obesidade apresentou alta adesão às práticas alimentares recomendadas pelo Guia, sendo as chances maiores entre os indivíduos idosos, com maior renda e melhor percepção de saúde. As práticas alimentares alinhadas às recomendações do Guia mais prevalentes foram comer sentado à mesa e a participação no preparo das refeições; e já entre as menos prevalentes a substituição do feijão por outras leguminosas, o uso de farinha de trigo do tipo integral e o hábito de tomar café da manhã.

A prevalência de alta adesão às recomendações do Guia foi superior à prevalência observada (23%) por Gabe e Jaime (14), ao investigarem uma amostra de conveniência das macrorregiões brasileiras. Essa diferença pode estar relacionada à inserção dos participantes deste estudo em um serviço de promoção da saúde, que objetiva promover modos de vida saudáveis, incluindo a alimentação adequada e saudável (16).

Apesar dos resultados positivos, é necessário intensificar as recomendações do Guia entre indivíduos com baixa e média adesão. Ao considerar que a adesão às práticas alimentares adequadas e saudáveis é influenciada por fatores individuais e ambientais, é crucial que os obstáculos para sua adoção, e os possíveis meios para superá-los (1) sejam trabalhados mediante intervenções estruturais e de educação alimentar e nutricional. Nesse sentido, a análise das dimensões da escala pode contribuir para identificar prioridades para intervenção. Neste estudo, a dimensão de planejamento foi a mais frágil entre os indivíduos com baixa adesão ao Guia, evidenciando a importância de trabalhar aspectos relacionados à compra de alimentos, cardápio semanal e compartilhamento das atividades domésticas, o que pode contribuir, inclusive, para reduzir os efeitos da falta de tempo, um dos principais obstáculos apontados pelo Guia (1). Também é necessário trabalhar a relevância do envolvimento com as refeições realizadas lar, da realização do café da manhã e de sentar-se à mesa (1). 

Adicionalmente, identificou-se reduzido uso de farinha integral, compra de alimentos em feiras-livres, e a diversificação do consumo de leguminosas para além do feijão. Ambos os aspectos podem se relacionar à menor renda dos participantes e a cultura alimentar local, que não favorece tais hábitos alimentares. Entretanto, pode-se reforçar a importância do consumo de outros alimentos ricos em fibras, como frutas e hortaliças, e a manutenção do consumo do feijão como principal componente do grupo das leguminosas visando a promoção da alimentação adequada e saudável.

A alta adesão às recomendações do Guia foi associada à fase da vida, como verificado por Gabe e Jaime (14). Essa relação pode estar atrelada aos modos de viver adotados com o aumento da idade e também a maior prevalência de doenças que demandam maiores cuidados com a alimentação e a saúde (17-19). Além disso, indivíduos idosos, em geral, possuem mais tempo disponível para se dedicar à compra/preparo dos alimentos e o exercício das habilidades culinárias (19).

Outro fator associado a alta adesão às recomendações do Guia identificado foi a renda familiar, importante determinante do acesso à alimentação adequada e saudável (20-21). Neste estudo, verificou-se que usuários no primeiro tercil de renda apresentaram menores chances de alta adesão às recomendações do Guia. Considerando que o entorno das unidades do Programa Academia da Saúde em Belo Horizonte, Minas Gerais apresentam menor densidade de estabelecimentos que comercializam alimentos saudáveis, limitações de renda e acesso podem ser dificultadores adicionais (22-23). Uma maior renda se associa a um maior poder de compra dos alimentos, principalmente diante do custo crescente dos alimentos in natura e minimamente processados no Brasil (24). Ademais, contribui para superar a oferta limitada desses alimentos no comércio local, por facilitar o acesso físico a alimentos com melhor qualidade e preço em outras regiões (25).

A alta adesão às recomendações do Guia também foi superior entre os participantes com autopercepção de saúde boa/muito boa. Por se tratar de um serviço de promoção da saúde, a participação no Programa pode percutir sobre adoção de comportamentos mais saudáveis e maior disposição, refletindo em uma percepção mais positiva da saúde e, consequentemente, na busca por mudanças na alimentação (26).

Apesar de seus resultados, este estudo apresenta limitações que devem ser consideradas. A sua realização em um serviço de promoção da saúde da APS, apesar de ser uma potencialidade, pode também limitar a inferência de seus resultados para a população geral. Usuários do Programa podem ser mais expostos a orientações alimentares realizadas por nutricionistas ou outros profissionais de saúde, além de serem majoritariamente mulheres (26). Tais aspectos podem repercutir sobre adesão ao Guia considerando que mulheres e indivíduos mais expostos a orientações sobre a saúde tendem a adotar mais comportamentos saudáveis, quando comparados às suas contrapartes. Todavia, 76% da população brasileira é coberta pela APS (27), e o país possui atualmente 3.437 unidades do Programa Academia da Saúde (28). Outra possível limitação foi a avaliação da adesão baseada na autopercepção (escala likert), que pode estar sujeita a viés de informação e de desejabilidade social. Para minimizar esses vieses, utilizou-se uma escala validada para a população brasileira (13).

Contudo, ressalta-se que este é o primeiro estudo a analisar o grau de adesão às práticas recomendadas pelo Guia entre usuários do SUS com obesidade, gerando contribuições importantes para o corpo de evidências. O uso de uma ferramenta validada, que mensura diferentes dimensões da alimentação adequada e saudável, em uma amostra representativa de serviço público de saúde, é igualmente inovador, permitindo uma avaliação abrangente das práticas alimentares dos participantes de um importante serviço da APS brasileira. Destaca-se, assim, o potencial deste estudo em contribuir para o aprimoramento das ações de implementação do Guia na APS, sobretudo entre pessoas com obesidade.

Conclui-se que a fase da vida, a melhor renda e a autoavaliação positiva da saúde foram fatores associados a alta adesão às práticas alimentares recomendadas pelo Guia entre usuários com obesidade do Programa Academia da Saúde de Belo Horizonte, Minas Gerais. Esses resultados evidenciam a necessidade de direcionar os esforços para o desenvolvimento de ações de promoção da alimentação adequada e saudável, pautadas no Guia, entre indivíduos adultos e pertencentes a grupos vulneráveis.
